# Development of Synergistic Antimicrobial Coating of *p*-Aramid Fibers Using Ag Nanoparticles and Glycidyltrimethylammonium Chloride (GTAC) without the Aid of a Cross-Linking Agent

**DOI:** 10.3390/polym9080357

**Published:** 2017-08-11

**Authors:** Chankyu Kang, Dajeong Ahn, Changhyun Roh, Sam Soo Kim, Jaewoong Lee

**Affiliations:** 1 Ministry of Employment and Labor, Major Industrial Accident Prevention Center, 10 Jeungheung 2ro Yeosusandallo, Yeosu-si 59615, Korea; chemnet75@korea.kr; 2 Department of Fiber and System Engineering, Yeungnam University, Gyeongsan 38541, Korea; andj1116@ynu.ac.kr; 3 Division of Biotechnology, Advanced Radiation Technology Institute (ARTI), Korea Atomic Energy Research Institute (KAERI), 1266, Sinjeong-dong, Jeongeup, Jeonbuk 56212, Korea; chroh@kaeri.re.kr

**Keywords:** silver nanoparticles, glycidyltrimethylammonium chloride, *p*-aramid fibers, antimicrobial activity, tensile strength

## Abstract

Functional *p*-aramid fibers that can express antimicrobial activity were produced by simple processing of silver nanoparticles (AgNPs), which are well known as antimicrobial agents, by using glycidyltrimethylammonium chloride (GTAC), a quaternary ammonium salt. *P*-aramid fibers were treated with GTAC by the pad-dry-cure process and put into an Ag colloid solution for reactions at 40 °C for 90 min to prepare GTAC/AgNPs-treated *p*-aramid fibers. Through these processes, GTAC was used as a substitute for existing cross-linking agents. The changes in the degree of attachment of AgNPs to the surface of *p*-aramid fibers were determined using a scanning electron microscope according to parameters such as GTAC concentration, Ag colloid concentration, and reaction temperature. Through this study, the following results were obtained: (i) The tensile strength of AgNPs/GTAC-treated *p*-aramid fibers was found to be about 80% of that of untreated *p*-aramid fibers; (ii) Thermogravimetric analysis showed that the thermal stability of *p*-aramid fibers did not change much after GTAC/AgNPs treatment and (iii) Antimicrobial activity analysis showed that AgNPs/GTAC-treated *p*-aramid fibers exhibited superior antibacterial properties compared to untreated *p*-aramid fibers, which may or may not be the effect of GTAC or AgNPs, or both.

## 1. Introduction

Along with the development of modern technology, super fiber polymers have been used in various fields in accordance with the trend toward lightweight materials with high functionality. In recent years, there has been a growing demand for a variety of functions of super fiber materials and accordingly, diverse studies of those materials have been in progress [1,2,3]. In particular, poly (*p*-phenylen terephthalamide) (PPTA, *p*-aramid), which has begun to be commercialized since the late 1970s, is a super fiber known by its product name, Kevlar. It has an arene substitution pattern in which functional groups face each other across aromatic hydrocarbons so that the skin part in the skin-core structure has higher crystal orientation than the core part [4,5]. With these structural properties, it is a high strength super fiber with tenacity not lower than 20 g/d, modulus in a range of 500~1100 g/d, heat resistance to endure at 400 °C, cold resistance to endure at low temperatures such as −160 °C, chemical resistance, and high energy absorption capacity [6,7], which is generally used for bulletproof vests, protective clothing, composite materials for aircraft and automobiles requiring high performance, and various industrial applications [5,6,7]. In particular, those *p*-aramid fibers that can be used for military and protective clothes do not have their own antimicrobial properties, and due to the nature of the fiber materials that have large surface areas, they have been exposed to the possibility of proliferation of microorganisms by contact with bacteria as a medium favorable for the growth of microorganisms. Since the majority of microorganisms are transferred from man to man by various fabrics, there is a growing need to improve antimicrobial properties as individuals become more interested in health and hygiene [8,9,10,11,12]. The production of antimicrobial fibers has increased significantly over the years. Ideally, the antimicrobial fibers should be effective against bacterial species and should not show any toxicity to the skin [13]. There are several methods for imparting antimicrobial properties to the fibers, such as placing the antimicrobial agent directly into the polymer, coating or attaching the antimicrobial agent on the surface of the polymer, and attaching the antimicrobial agent through chemical or physical bonding [14].

The use of antimicrobial nanoparticles (NPs) is one of the promising strategies to overcome the drug resistance of microorganisms [15]. NPs have unique physical and chemical properties that are different from bulk materials based on properties such as size, distribution, and morphology [16,17,18,19,20]. Currently, the utilization of fibers applied with silver nanoparticles (AgNPs) has been increasing compared to those applied with inorganic nano-particles and studies of antimicrobial functionality have been coming to the fore with very high importance in modern life where hygiene and health are considered important [21,22]. Silver has been considered as a disinfectant [23], and AgNPs, in particular, have been reported to have excellent antibacterial properties [24]. Their biological properties vary depending on the metal size, structure, and surface area of the nanometer particles [25]. Since there are difficulties in effectively attaching AgNPs to *p*-aramid fibers, crosslinking agents are needed to solve this problem and effectively connect AgNPs. For example, binding of AgNPs to cotton using butane tetracarboxylic acid [26], binding of AgNPs to carbon fibers using chitosan [27], and binding of AgNPs to cotton using 3-mercaptopropyltrimethoxysilane (3-MPTMS) preceded this study [28]. Thus, 3-MPTPMS has been widely used to provide functional groups specific to AgNP as well as other types of nanoparticles [29,30].

Meanwhile, quaternary ammonium salt (QAS), which is composed of nitrogen atoms bonded to four carbons per atom, is strongly positively charged thereby strongly attracting negatively charged bacteria to contact with them and the long aliphatic alkyl chains of QAS are known to kill bacteria. Previous studies have demonstrated the effectiveness of methods of imparting antibacterial properties by attaching QAS to a substance [31,32]. Glycidylmethylammonium chloride (GTAC) is a kind of QAS, which structurally contains epoxy rings that enable chemical bonding with –OH, –NH_2_, and –SH groups through reactions [33]. In addition, GTAC has considerable affinity to water due to the presence of the –N^+^(CH_3_)_3_ group [34], and is a QAS that receives much attention as an antibacterial agent [14].

In the previous study, AgNPs were tested for antimicrobial activity against a natural fiber, such as cotton fibers, using GTAC and 3-mercaptopropyltrimethoxysilane (3-MPTMS), a cross-linking agent [35]. However, it is necessary to develop ways to minimize the inconveniences caused by using these catalysts while giving similar antimicrobial effects. The purpose of this study was to develop a new method without the aid of cross-linking agent 3-MPTMS, and to introduce applicable techniques of AgNPs to *p*-aramid fibers, a kind of synthetic fiber. The physical and antimicrobial properties of *p*-aramid fibers after the reaction of AgNPs and GTAC were investigated. At this time, GTAC acts as an alternative agent without 3-MPTMS used as a cross-linking agent to confirm that AgNPs can attach to *p*-aramid fibers. Diverse conditions, such as GTAC concentration, Ag colloid concentration, and reaction temperature, were used to verify the increased antimicrobial activity of GTAC/AgNPs-treated *p*-aramid fibers.

## 2. Materials and Methods

### 2.1. Materials

Areal density of *p*-aramid fabric was 155 g/m^2^ and provided by the Kolon Industries (Gumi, South Korea). The *p*-aramid fabric was refined before being used in this study. In the *p*-aramid fabric refining process, the *p*-aramid fabric was washed twice with distilled water at 60 °C, sufficiently washed twice with ethanol and acetone, respectively, at room temperature thereafter, and naturally dried for 24 h. The GTAC (90% Sigma-Aldrich, St. Louis, MO, USA) used to impart antimicrobial properties and silver colloid (Nanomix-silver 30,000 ppm, Seoul, Korea) were used without any further purification.

### 2.2. Manufacture of QAS Treated p-Aramid Fibers

In order to treat *p*-aramid fibers with a GTAC solution, which is a sort of QAS, fibers were prepared with 0, 10, 20, 30, and 40 wt % concentrations using distilled water as a solvent. The pad-dry-cure process was used for the preparation of samples and the concrete method, as follows. The *p*-aramid fabric was immersed in the GTAC solution for 30 min at ambient temperature without stirring. Thereafter, the treated samples were preliminarily dried at 80 °C for 30 min, while maintaining the wet-pick up ca. 100%, cured at 190 °C for 15 min, and then washed with distilled water to remove unattached GTAC, followed by drying naturally at ambient temperature.

### 2.3. Manufacture of AgNPs-Coated p-Aramid Fibers

To coat *p*-aramid fibers with AgNPs, untreated and GTAC-treated samples were immersed in a solution prepared by adding silver colloid in a constant temperature water bath and the solution was stirred for 90 min at 110 rpm at 40 °C. Thereafter, the samples were rinsed with distilled water twice to remove unattached AgNPs, and dried naturally thereafter.

### 2.4. Transmission Electron Microscope (TEM)

A transmission electron microscopy (H-7600, Hitachi, Tokyo, Japan) was used with an acceleration voltage of 120 kV to observe the size and dispersion state of AgNPs.

### 2.5. Scanning Electron Microscope-Energy Dispersive Spectroscopy (SEM-EDS)

The surface characteristics of the treated *p*-aramid fabric and the elements attached to the surface were analyzed using SEM-EDS (S-4100, Hitachi, Tokyo, Japan). The samples were coated with thin films of platinum before the analysis and the surface of the fabric was observed under an acceleration voltage of 15 kV. To compare and analyze the component characteristics of the in-depth surface composing elements of the pure *p*-aramid fibers, *p*-aramid fibers treated with the GTAC or AgNPs alone, and those complex treated with GTAC/AgNPs, the surface of each of them was observed at a magnification of ×3.00 K using energy dispersive element analysis (EDS). The operating mode of the SEM was SE. In order to measure Ag content attached on the *p*-aramid fibers, Ag (atomic %) under several factors, such as GTAC concentration, Ag colloid concentration, treatment temperature, and washing cycles, was measured with EDS.

### 2.6. Washing Fastness

The washing fastness was evaluated to determine the durability of AgNPs bound to *p*-aramid fibers. The washing fastness test was conducted using the test method under KS K IOS 105-C01: 2007. This method was implemented using cylinders made of glass or stainless steel with a diameter of (75 ± 5) mm, a height of (125 ± 10) mm, and a volume of (550 ± 50) mL. A detergent aqueous solution was prepared at 5 g/L using AATCC standard detergent WOB (without optical brightener). The soap solution prepared as such was put in the cylinders at a solid/liquid ratio of 1:50 to wash the test pieces at a temperature of (40 ± 2) °C for 30 min at a rotation speed of 40 ± 2 min^−1^. The test pieces were taken out after the washing process, washed twice with distilled water, and dried at ambient temperature before being observed.

### 2.7. Tensile Strength

The mechanical properties of *p*-aramid fibers treated with GTAC and AgNPs were evaluated using the test method of ASTM D 7269. Before the analysis, 100 ± 2 mm wide, 150 mm long test specimens were prepared in accordance with the test specifications. The tensile tester was set to have a gripping distance of 75 ± 1 mm and an elongation velocity of 300 ± 10 mm/min. The test specimen was gripped so that the alignment line on the test specimen came into contact with the sides of the 25 mm wide front jaws on the top and bottom, and then placed on a straight line before the test.

### 2.8. Thermogravimetric Analysis (TGA)

To analyze the thermal properties of untreated *p*-aramid fibers and GTAC and Ag colloid treated *p*-aramid fibers, TGA weight loss curves, which are thermal analysis data, were analyzed using TG-DTA (SDT Q600, TA Instruments, New Castle, DE, USA). The measurement was conducted in the N_2_ purge state while the temperature was rising at a rate of 10 °C/min up to 600 °C.

### 2.9. Antimicrobial Activity Analysis

The strains used for the antimicrobial test were *Escherichia coli* O157: H7 (ATCC 43895) (Gram-negative bacteria), *Pseudomonas aeruginosa* PAO1 (ATCC 15692) (Gram-negative bacteria), and *Staphylococcus aureus* MRSA (ATCC BAA-1707) (Gram-positive bacteria). All of them were received from Korean Culture Center of Microorganisms (Seoul, South Korea) and grown into Luria-Bertani agar (BD biosciences, Franklin Lakes, NJ, USA) broth for 37 °C at 18 h, were inoculated into the fabric. A 1 × 1 cm^2^ sized fabric sample was inoculated with 1 mL of the activated bacteria in the liquid medium, put into 0.85% physiological saline 24 h later, and vortexed. Thereafter, the bacterial concentration was adjusted using the decimal system and the diluted solution was used in the test. Antimicrobial activity was tested using the disc diffusion method (Halo test), which is a qualitative test, and the AATCC Test Method 100 (Contact assay in liquid), which is a quantitative test. A non-ionic surfactant, Triton X-100 (Sigma-Aldrich, St. Louis, MO, USA), was used to effectively put the *p*-aramid fiber sample into contact with the solid medium while conducting the Halo test.

## 3. Results and Discussion

### 3.1. Surface Analysis of GTAC- and AgNPs-Treated Samples

**Figure 1:**
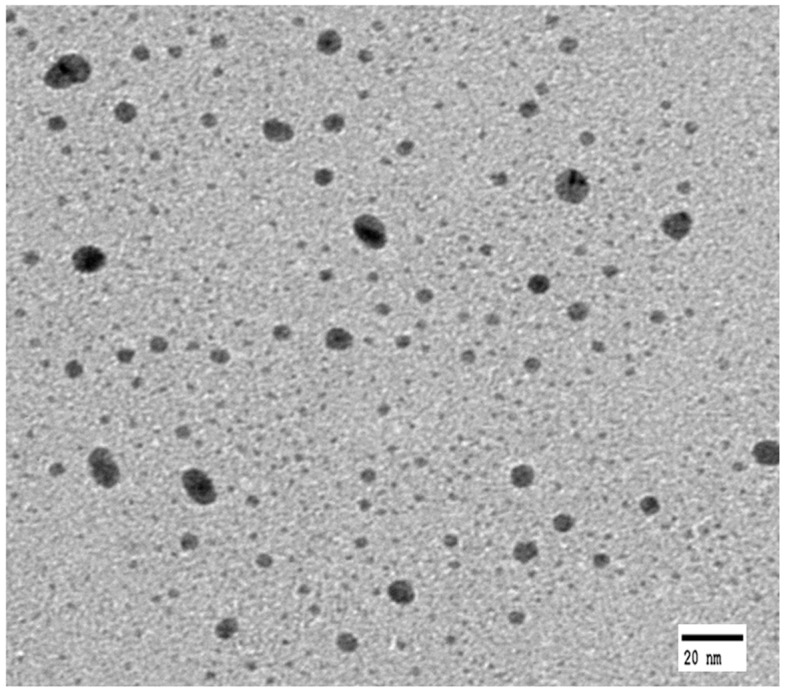
Transmission electron microscopy (TEM) image of AgNPs in the silver colloid solution.

**Figure 2:**
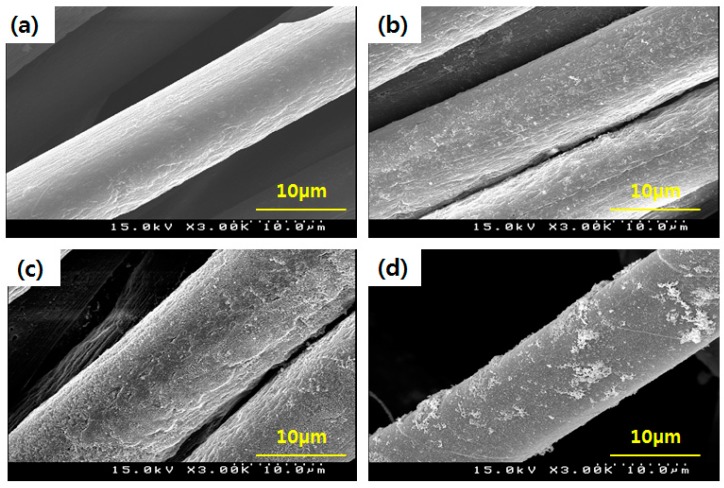
Scanning electron microscopy (SEM) micrograph of (**a**) untreated *p*-aramid fiber and (**b**) treated *p*-aramid fiber with 10 wt % of GTAC/2500 ppm of AgNPs; (**c**) treated *p*-aramid fiber with 10 wt % of GTAC/10,000 ppm of AgNPs; and (**d**) treated *p*-aramid fiber with 10 wt % of GTAC/30,000 ppm of AgNPs.

**Figure 3:**
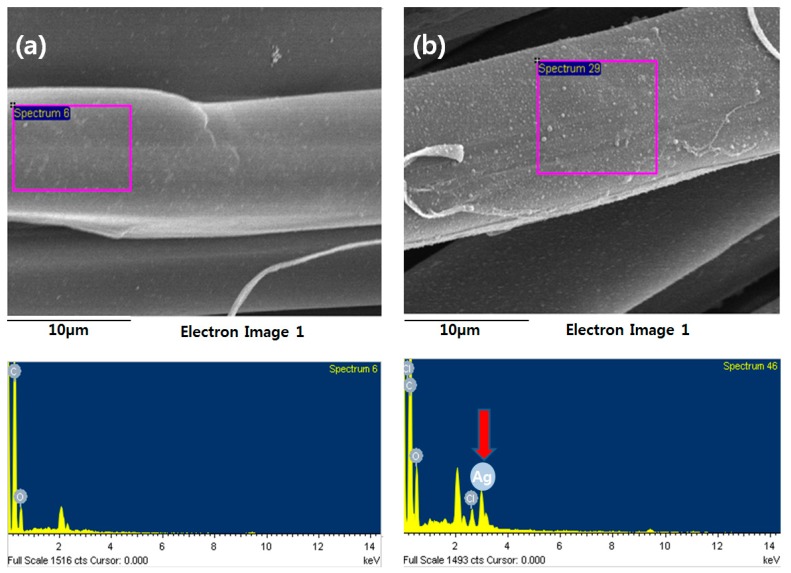
SEM-EDS peak of (**a**) untreated *p*-aramid and (**b**) treated *p*-aramid with 30,000 ppm of AgNPs/10 wt % of GTAC.

**Figure 4:**
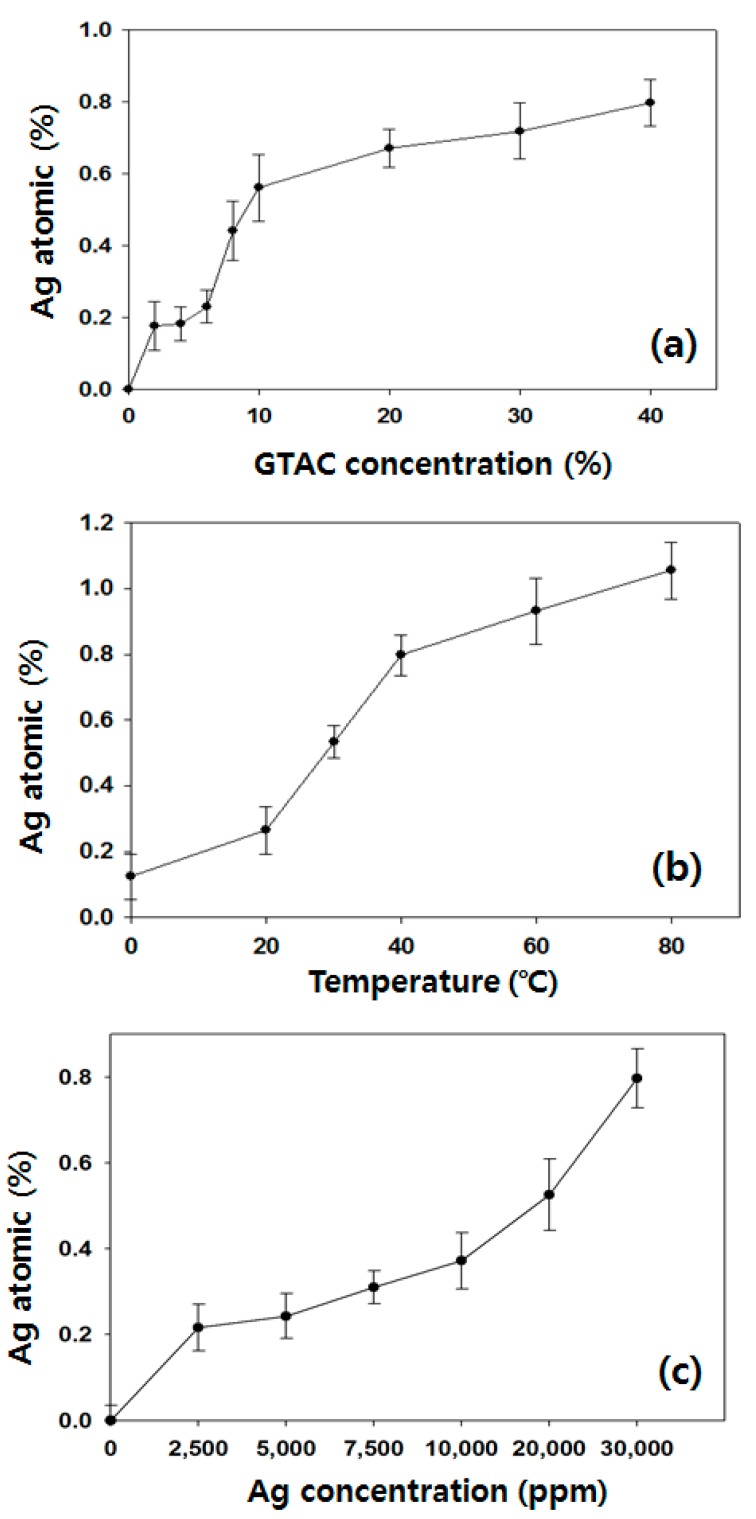
Ag (atomic %) on GTAC/AgNPs treated *p*-aramid under different (**a**) GTAC concentration; (**b**) temperature; and (**c**) Ag colloid concentration.

The TEM images of AgNPs dispersed in a silver colloid solution are shown in Figure 1, taken before attaching AgNPs to the *p*-aramid fiber surface in order to identify the sizes and distribution of the AgNPs dispersed in a silver colloid solution. The AgNPs in the silver colloid were found to be relatively uniformly dispersed, and the sizes of the observed were 1 ~ 20 nm. The mean size of the observed AgNPs was 11.43 ± 2 nm. The stabilized AgNPs without agglomeration were found in solution, indicating that bare AgNPs were involved in the reaction. This is because the asymmetrical charge distribution of AgNPs is minimized by dissolved water, which prevents aggregation of the AgNPs [36]. Figure 2 showed the surface shapes of pure *p*-aramid fibers and GTAC/AgNPs-treated *p*-aramid fibers observed using a SEM at a magnification of ×3.00 K in order to compare the surface roughness of pure *p*-aramid fibers and GTAC/AgNPs-treated *p*-aramid fibers. The untreated *p*-aramid fibers had relatively smooth surfaces (see Figure 2a), while the *p*-aramid fibers treated with GTAC and AgNPs had somewhat rougher surfaces as the concentration of the GTAC and AgNPs increased (see Figure 2b). When treated with AgNPs at concentrations exceeding 10,000 ppm, the surfaces of the *p*-aramid fibers showed remarkable changes because of the chemical bonding between the functional groups of the AgNPs and the fiber surface (Figure 2c). However, when the concentration of AgNPs exceeded 30,000 ppm, the changes of the surface were slowed because the existed functional groups on the surface were sufficiently bonded to AgNPs and did not allow any further bonding (Figure 2d). The results of analysis of chemical composition changes with increasing surface roughness are shown in Figure 3. It showed the results of measurement of the elemental components of untreated *p*-aramid fibers and *p*-aramid fibers treated with GTAC and AgNPs using SEM/EDS for comparison and analysis of the chemical structures. Peaks of Ag and Cl groups, which were not seen in untreated *p*-aramid fibers, were observed in the *p*-aramid fibers treated with GTAC and AgNPs. Based on these results, it indicated that the chemical reaction of AgNPs and GTAC effectively attached to *p*-aramid fibers. In addition, the surface coating of the AgNPs attached to the *p*-aramid fibers exhibited a high degree of homogeneity, as shown in Figure 3b. Studies on the factors affecting the reactivity of silver nanoparticles on the surface of *p*-aramid fibers were analyzed in Figure 4. The effects of GTAC concentrations on Ag (atomic %) of *p*-aramid fibers are shown in Figure 4a. As the treatment concentration of GTAC increased, Ag (atomic %) attached to *p*-aramid fibers increased. In particular, Ag (atomic %) increased rapidly until 10 wt % GTAC, and the increasing rate gradually decreased thereafter. This was assumed to be because the number of amine groups (–NH_2_) existing at the end of the *p*-aramid polymer chain which can chemically react with GTAC was limited to a certain range even if the treatment concentration of GTAC used as a crosslinking agent increased. The Ag (atomic %) of *p*-aramid fibers measured according to the treatment temperature is shown in Figure 4b. It means that the reaction temperature is an important factor. When the reaction temperature reached 40 °C, AgNPs effectively bound to the *p*-aramid fibers. This was probably due to the increased mobility of AgNPs, resulting in AgNPs being more aggressively mitigated and reacting with GTAC. Therefore, AgNPs attached to the surface of *p*-aramid fibers were increased. Figure 4c shows changes in Ag (atomic %) on *p*-aramid fibers measured according to the concentration of Ag colloid. As the Ag colloid concentration increased, Ag (atomic %) increased linearly. Small amounts of Ag (atomic %) on *p*-aramid fibers were identified through measurement. This was probably due to the dense nature of the structure of *p*-aramid fibers, which limited reactions to occurring between reactive sites on *p*-aramid fibers and GTAC/AgNPs.

### 3.2. Physical and Thermal Properties

The tensile strength analyses of *p*-aramid fibers before and after treatment with GTAC/AgNPs were measured and the results are shown in Table 1. The tensile strength of GTAC-treated *p*-aramid fibers and GTAC/AgNPs-treated *p*-aramid fibers decreased by 19% and 20%, respectively, compared to untreated *p*-aramid fibers. However, no decrease in tensile strength was observed when AgNPs reacted with *p*-aramid fibers. This was probably due to the fact that the Cl^−^ existing in GTAC affected the physical properties of the *p*-aramid fibers. Previous studies reported that the *p*-aramid fibers have a significantly lower tensile strength on exposure to chlorine-containing sodium hypochlorite [35].

**Figure 5:**
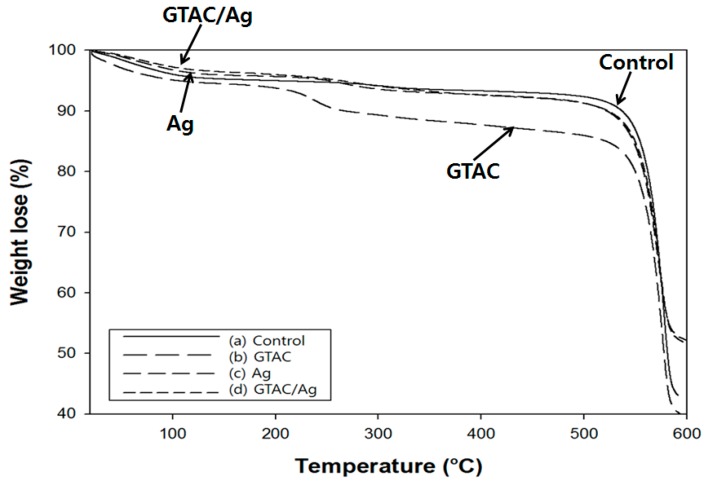
TGA pattern of (**a**) untreated *p*-aramid and (**b**) treated *p*-aramid with 10 wt % of GTAC; (**c**) treated *p*-aramid with 30,000 ppm of AgNPs; (**d**) treated *p*-aramid with 10 wt % of GTAC/30,000 ppm of AgNPs.

The thermograms of *p*-aramid fibers, including untreated *p*-aramid fibers, GTAC-treated *p*-aramid fibers, and *p*-aramid fibers complex-treated with GTAC and AgNPs at different concentrations are shown in Figure 5. In the *p*-aramid fibers treated with GTAC, AgNPs, and GTAC/AgNPs, the reduction of initial degradation temperature was the same as that of untreated *p*-aramid fibers. However, two weight-loss curves were found at 220 and 540 °C, respectively. The small curve observed at 220 °C was estimated to be due to the pyrolysis of GTAC. When GTAC was treated together with AgNPs, no curve was observed at 220 °C, indicating that the pyrolysis of GTAC was prevented by AgNPs to some extent.

### 3.3. Durability and Antimicrobial Efficacy

The washing fastness was measured to evaluate the durability of AgNPs attached to the surface of *p*-aramid fibers treated with GTAC/AgNPs, and the results are shown in Table 2. After five washing cycles, the maintenance rate of Ag (atomic %) was observed to be about 34% compared to that without washing. This result was associated with the material properties of *p*-aramid fibers, such as the straight orientation, the rigid structure with high crystallinity, and the relatively small portion of amorphous regions. Therefore, GTAC/AgNPs did not completely penetrate *p*-aramid fibers and weakened the durability of AgNPs by washing. After 30 consecutive laundering cycles, the Ag content almost disappeared, but the bacterial reduction rates against tested bacteria remained above 95% over 10 cycles. The antimicrobial property shows that the durability of AgNPs against washing cycles is lower than that of using a chitosan derivative binder for cotton [37], but the use range of *p*-aramid will be extended if it combines the superiority and antibacterial property of *p*-aramid fibers.

**Figure 6:**
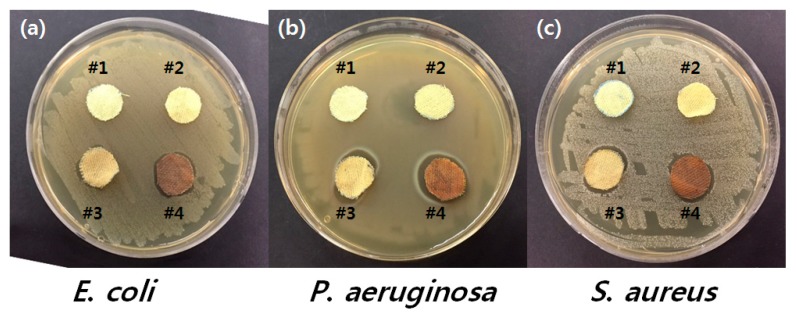
Antimicrobial activity of #1 untreated *p*-aramid, #2 *p*-aramid treated with GTAC, #3 *p*-aramid treated with AgNPs, and #4 *p*-aramid treated with GTAC/AgNPs.

The antimicrobial properties of the untreated *p*-aramid fibers and the treated *p*-aramid fibers were compared and the results are shown in Figure 6 and Table 3. The results of the Halo test, one of the qualitative methods, shown in Figure 6, visually present the degree of the formation of the zones of inhibition around the sample. After placing the sample on the culture medium smeared with bacteria, the zones of inhibition appearing in sample #4 complex-treated with GTAC/AgNPs can be identified. To verify antibacterial activity with different cases, the samples were put into contact with bacteria for a certain period of time and the zones of inhibition were measured. The results are shown in Table 3. In the case of *E. coli*, inhibition of bacteria was observed in samples treated with AgNPs or GTAC/AgNPs. However, in *P. aeruginosa*, which is known to have strong multi-drug resistance as a kind of Gram-negative bacteria [38], a single antimicrobial substance (i.e., GTAC or AgNPs) exhibited relatively good antimicrobial properties as compared to untreated *p*-aramid, but was not completely inhibited. On the contrary, *P. aeruginosa* was effectively inactivated in sample #4 simultaneously treated with both GTAC/AgNPs. *S. aureus* MRSA bacteria, as shown in Table 3, exhibited low levels of antimicrobial activity. In sample # 2 and sample #3, a relatively large number of residual bacteria were found using a single antimicrobial substance of GTAC or AgNPs [12,39]. The combination of GTAC and AgNPs instead of a single antimicrobial substance resulted in inhibition of *S. aureus* and did not detect the corresponding bacteria in sample #4. Consequently, it was identified that synergistic antimicrobial activity could be expressed when *p*-aramid fibers were treated with both GTAC and AgNPs. Furthermore, this synergistic antimicrobial activity was retained after five washing cycles (see Table 3).

## 4. Conclusions

AgNPs were effectively attached to *p*-aramid fibers using GTAC, a quaternary ammonium salt, without using conventional cross-linking agents. Through SEM surface analysis, it was identified that the surface of the *p*-aramid fibers became relatively rough after the treatment with GTAC/AgNPs. It was found that several factors play a role in the surface property change through the increase in the Ag (atomic %) of *p*-aramid fibers because of the increase of the treatment temperature and the concentration of GTAC and AgNPs. Compared to *p*-aramid fibers treated with GTAC or AgNPs alone, *p*-aramid fibers treated with a combination of GTAC/AgNPs exhibited synergistic antibacterial properties. The GTAC/AgNPs-treated *p*-aramid fibers, which exhibit increased antimicrobial activity without significantly altering the existing physical properties of *p*-aramid fibers, can be used as complex functional fibers. These *p*-aramid fibers exhibit excellent antimicrobial performance and are believed to be useful in a variety of applications to protect the human body and industrial textile fields, including textiles for infection prevention.

## Figures and Tables

**Table 1:** Tensile strength of untreated and GTAC/AgNPs treated *p*-aramid.

Control (Treated with Distilled Water)	Treated with GTAC (10 wt %)	Treated with AgNPs (30,000 ppm)	Treated with GTAC (10 wt %)/AgNPs (30,000 ppm)
33.06 ± 0.69	26.75 ± 1.40	27.23 ± 1.23	26.23 ± 0.84

**Table 2:** Washing fastness of GTAC/AgNPs-treated *p*-aramid.

Washing (Cycles)	Ag (Atomic %)	Retention (%)
0	0.79	100
1	0.63	79.75
2	0.48	60.76
3	0.42	53.16
4	0.33	41.77
5	0.27	34.18

**Table 3:** Antimicrobial test results of untreated and treated *p*-aramids.

Samples	Bacterial No. (cfu/Sample)		
	*Escherichia coli* ^a^	*Pseudomonas aeruginosa* ^b^	*Staphylococcus aureus* ^c^
Untreated *p*-aramid (# 1)	2.9 × 10^5^	4.2 × 10^5^	1.34 × 10^5^
*p*-aramid treated with GTAC (# 2)	1.18 × 10^2^	4.65 × 10^3^	5.05 × 10^2^
*p*-aramid treated with AgNPs (# 3)	0	1.7 × 10^2^	3.24 × 10^3^
*p*-aramid treated with GTAC/AgNPs (# 4)	0	0	0
*p*-aramid treated with GTAC/AgNPs after 5 washing cycles	0	0	0

**^a^** Total bacteria: 5.7 × 10^6^ cfu/sample; **^b^** Total bacteria: 8.9 × 10^6^ cfu/sample; **^c^** Total bacteria: 7.6 × 10^6^ cfu/sample.
